# JH-2 constitutive model of sandstone for dynamic problems

**DOI:** 10.1038/s41598-023-49668-z

**Published:** 2024-02-09

**Authors:** Paweł Baranowski, Michał Kucewicz, Jacek Janiszewski

**Affiliations:** Institute of Mechanics and Computational Engineering, Faculty of Mechanical Engineering, Military University of Technology, 2 Gen. S. Kaliskiego Street, 00-908 Warsaw, Poland

**Keywords:** Mechanical properties, Mechanical engineering

## Abstract

This paper demonstrates the application of the Johnson–Holmquist II (JH-2) model with correlated and validated parameters to simulate the behavior of a sandstone. The JH-2 model is used to simulate various tests, including single-element tests, structural quasi-static uniaxial and triaxial compression tests, and the split Hopkinson pressure bar test. Additionally, the model is used to simulate drop-weight impact test using a ball bearing and two loading scenarios involving small-scale blasting and projectile impacts. Quantitative and qualitative comparisons demonstrate that the JH-2 model agrees well with both experimental and analytical results. Limitations of the model are also highlighted, particularly for quasi-static problems, as the model was originally developed for high-strain-rate simulations. Ultimately, this study demonstrates that the JH-2 rock constitutive model can obtain reasonable results for a material other than the material for which the model was originally correlated and validated. This paper provides valuable guidance for modeling and simulating sandstone and other rock materials subjected to dynamic loadings.

## Introduction

A high-fidelity constitutive model is required to reproduce the behavior of rocks under dynamic conditions, such as impacts and blasts. The model should capture as many physical properties of the simulated rock as possible, including pressure dependency, strain-rate effects, softening, damage evolution and shear dilatancy. Several constitutive models have been developed; most are designed for modeling concrete, but some are dedicated exclusively to rock. The models most commonly used to simulate blasting and projectile penetration problems are the Riedel–Hiermaier–Thoma (RHT)^1–5^, Karagozian and Case Concrete (KCC)^6–8^ and Continuous Surface Cap (CSC) models^9,10^. The Johnson–Holmquist Concrete (JHC)^11–14^ and Johnson–Holmquist ceramics (JH-2) models^15–23^, which were proposed by Timothy Johnson and Gordon Holmquist^24^, the brittle damage model^25,26^, and the Taylor-Chen-Kuszmaul (TCK) model^27^, among others^28,29^, are also used in the literature.

Some of the abovementioned constitutive models were used to extensively study the dynamic behavior of sandstone. These studies have included medium- and large-scale^30–32^ fracture and cracking experiments, tunneling simulations^33–36^, small-scale blasting tests^37,38^, and different impact scenarios^39–43^. For instance, the RHT constitutive model was used to simulate penetration and perforation tests of red sandstone and limestone^43^ and satisfactorily reproduced the blasting and sandstone fracture mechanism around a tunnel-shaped cavity^35^. The same approach was used to analyze the influence of blasting load directions on tunnel stability^36^. The RHT material model was also used to estimate the spalling strength of sandstone under different pre-confining pressures^44^. The JHC model was effectively used to study the characteristics of rock damage in decoupled charge blasting-based numerical simulations combined with experiments and computed tomography scanning^45^. After determining the parameters of the JHC model for sandstone, the model was used to analyze specimen damage under single and repeated impacts in the split Hopkinson pressure bar (SHPB) test^46^. A simple model based on the Concrete Damage Plasticity (CDP) model was used to analyze internal blasts in a rock tunnel^34^. To analyze sandstone damage behavior under blasting loading conditions using FLAC3D code, sandstone was described using the Mohr–Coulomb failure criterion^30^. A novel constitutive model with plastic internal and damage variables for brittle rock was proposed and validated through a series of laboratory tests^47^. Small-scale blasting with pre-cracks was combined with the material model based on major principal stress and the maximum shear stress failure criterion to analyze the effect of crack length on mode I crack propagation^37^. Blast-induced damage in tunnels was modeled with a hybrid finite-discrete numerical approach using the fracture energy approach controlled by designated constitutive fracture criteria^33^. A dynamic bounding surface plasticity damage model was proposed for rocks subjected to high strain rates and confinements^48^. Other interesting studies of sandstone modeling and simulations are also available in the literature^49,50^.

The JH-2 model is very effective and has been widely used in studies of blasting, impacts and other strongly dynamic scenarios^15–21,51^. Quite recently, modifications of the model to improve its deficiencies in the numerical reproduction of single- and dual-borehole blasting were proposed^52^. A user-defined modification of the model was then verified through small-scale blast tests and used to simulate borehole blasting^19^. Notably, few papers have used the JH-2 constitutive relation for the numerical reproduction of sandstone, thus, it was undertaken in this paper. For instance, a model using parameters determined for red sandstone was validated by reproducing an SHPB test, but the model was not further verified in different loading scenarios^53^. The JH-2 model was also used with sandstone to analyze blast effects near tunnels^54^; however, the majority of the adopted parameters were for limestone. A similar study did not discuss the determination of the sandstone parameters used in the numerical simulations^55^. The advantages and disadvantages of this model compared to other material models were discussed in the authors’ previous paper^56^. Therefore, a short clarification of the selection of the JH-2 model is provided in this section.

Deficiencies of the JH-2 model have been noted, particularly its inability to reproduce shear dilatancy, strain-hardening and Lode-angle effects. Moreover, despite that tensile and shear/compression damage mechanisms are included, it is not possible to separate tensile and shear/compression damage in the presentation of the results since a single scalar damage parameter *D* is used. The JH-2 model has incorrectly implemented tensile damage in a pure tension, which was also noted in the present paper. To overcome these limitations, user-defined modifications of the model can be introduced^52,57,58^.

On the other hand, there are several advantages of this model compared to more sophisticated models such as RHT, CSC, user-defined, etc. First, a calibration can be conducted based only on the basic tests, and the other parameters can be obtained using an iterative approach based on experimental results. Second, the JH-2 model can be relatively easy implemented because there is no need for using the FORTRAN subroutines procedures required for user-defined material models. Furthermore, a high accessibility of source data/results and numerous studies confirming the accuracy and feasibility of this model are other factors that led to its selection in the present paper. Despite its drawbacks, the original JH-2 model can faithfully reproduce rock behavior under various loading conditions within certain ranges of tolerance and reliability, which was also demonstrated in the present paper.

Based on the above, the main aim of the present paper was to provide parameters for the JH-2 model determined for a sandstone by using numerical and experimental approaches. The scientific and novel aspects are as follows: (1) implementation of this model for reproducing a sandstone behavior; (2) correlation and validation of the JH-2 constitutive model based on several tests to validate its fidelity under various stress and strain-rate conditions and (3) demonstration of the model effectiveness in the projectile impact into a different sandstone target. In all cases, a satisfactory quantitative and qualitative agreement with the experimental and analytical results was obtained.

## Sandstone description

Specimens of sandstone were acquired from material cores from the Lower Silesia region in Poland. To characterize the basic mechanical properties of the investigated sandstone, a series of laboratory tests was conducted in quasi-static and dynamic loading regimes. Most of the tests were carried out according to the International Society for Rock Mechanics (ISRM) standards for rock testing^59^. A detailed description of the tests is not included in the present paper. The basic mechanical properties are listed in Table 1.

**Table 1:** Physical and mechanical properties of the studied sandstone (average).

Experimental test	Parameter	Value	Unit
–	Density, *ρ*	2350.0	kg/m^3^
–	Poison’s ratio, *v*	0.21	–
–	Bulk modulus, *K*_*1*_	3735.0	MPa
–	Shear modulus, *G*	2687.0	MPa
Uniaxial compression test	Elastic modulus^a^, *E*	6510.0	MPa
	Static uniaxial compressive strength^a^, *UCS*_*S*_	79.1	MPa
Brazilian static test	Static uniaxial tensile strength^a^, *UTS*_*S*_	2.2	MPa
Brazilian dynamic test	Dynamic uniaxial tensile strength^a^, *UTS*_*D*_	14.0	MPa
Triaxial test, *σ*_2_ = *σ*_3_ = 10 MPa	Triaxial compressive strength^a^, *TCS*_1_	102.5	MPa
Triaxial test, *σ*_2_ = *σ*_3_ = 17 MPa	Triaxial compressive strength^a^, *TCS*_2_	123.4	MPa
Triaxial test, *σ*_2_ = *σ*_3_ = 25 MPa	Triaxial compressive strength^a^, *TCS*_3_	159.2	MPa
SHPB test, $\dot{\varepsilon }$ = 80.0 s^−1^	Dynamic uniaxial compressive strength^a^, *UCS*_*D*1_	94.1	MPa
SHPB test, $\dot{\varepsilon }$ = 110.0 s^−1^	Dynamic uniaxial compressive strength^a^, *UCS*_*D*2_	105.2	MPa
SHPB test, $\dot{\varepsilon }$ = 240.0 s^−1^	Dynamic uniaxial compressive strength^a^, *UCS*_*D*3_	123.4	MPa

^a^Averaged values from a series of specimens.

## Determination of JH-2 model parameters

### Brief JH-2 description

**Figure 1:**
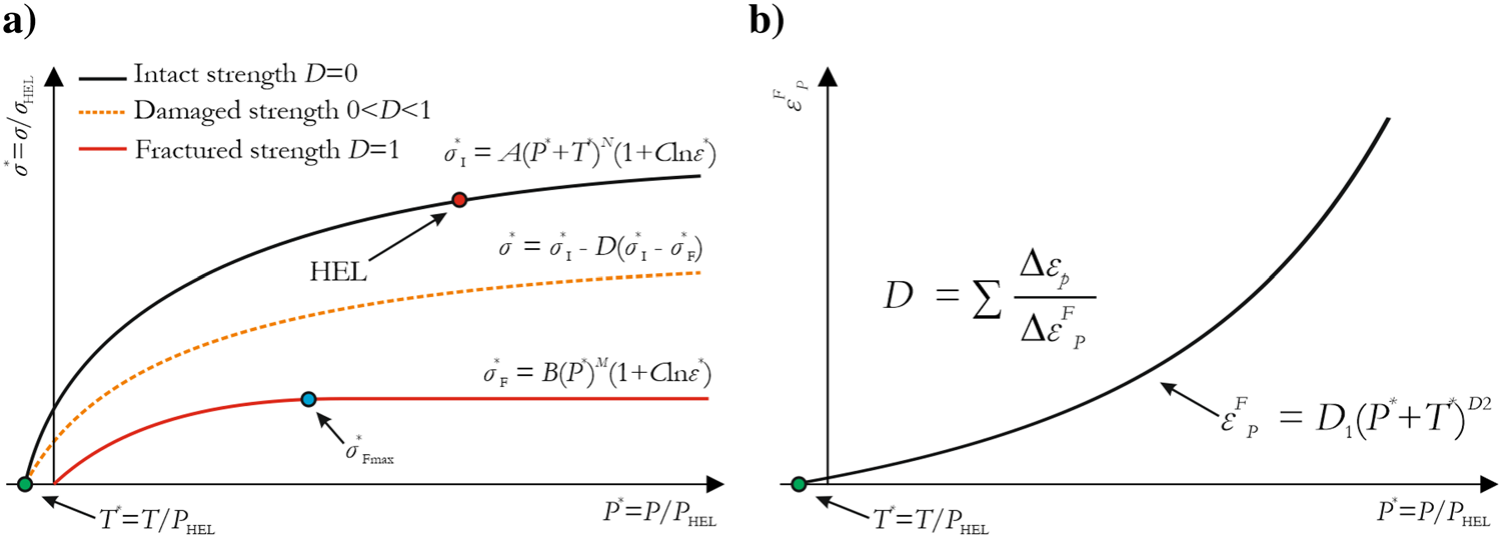
(**a**) Intact, damaged and fractured surfaces of the JH-2 constitutive model; (**b**) characteristic of the equivalent fracture plastic strain versus normalized pressure, which describes the accumulation of damage in the model identified using two constants: *D*1 and *D*2.

Since details of the JH-2 model can be found in several papers, only a brief description of the model is given in the present paper. The JH-2 model describes the relationships between normalized pressure and normalized equivalent stress for intact, damaged, and fractured surfaces (Fig. 1a). The normalized intact strength (black line) of the material is described using the following formula^15,16,52,60^:

1 $$ \sigma *_{I} = A\left( {P* + T*} \right)^{N} \left( {1 + C \cdot \ln \mathop \varepsilon \limits^{.} *} \right) $$

where $\sigma *_{I} = \sigma_{I} /\sigma_{HEL}$ is the normalized intact equivalent strength ($\sigma_{I}$ is the current equivalent stress, and $\sigma_{HEL}$ is the equivalent stress at the Hugoniot elastic limit (HEL)); $P* = P/P_{HEL}$ is the normalized hydrostatic pressure (*P* is the current hydrostatic pressure); $T* = T/P_{HEL}$ is the normalized maximum tensile hydrostatic pressure; *C* is a coefficient defining the intensity of strain rate enhancement; and $\dot{\varepsilon }* = \dot{\varepsilon }/\dot{\varepsilon }_{0}$ is the dimensionless strain rate ($\dot{\varepsilon }$ is the current equivalent strain rate, and $\dot{\varepsilon }_{0} = 1.0\,s^{ - 1}$ is the reference strain rate).

When damage accumulates, the material strength decreases through iterative decreases in the damage surface $\sigma *_{D}$, which is represented by the dashed line in Fig. 1a and is calculated as follows^15,16,52,60^:

2 $$ \sigma *_{D} = \sigma *_{I} - D\left( {\sigma *_{I} - \,\,\sigma *_{F} } \right) $$

where *D* is a damage factor with a value between 0 (undamaged) and 1 (fully damaged). In this state, the material is partially damaged (0 ≤ *D* ≤ 1).

As the plastic deformation increases, the material becomes fully fractured, and the normalized fractured strength of the material, represented by $\sigma *_{F}$, is described by the following equation^15,16,52,60^:

3 $$ \sigma *_{F} = B\left( {P*} \right)^{M} \left( {1 + C \cdot \ln \dot{\varepsilon }*} \right) $$

where *B* and* M* are the fractured material constants.

The damage in an infinite volume is calculated as follows^15,16,52,60^:

4 $$ D = \sum {\frac{{\Delta \varepsilon_{P} }}{{\varepsilon_{P}^{F} }}} $$

where $\Delta \varepsilon_{P}$ is the increment of the equivalent plastic strain and $\varepsilon_{P}^{F}$ is the equivalent fracture plastic strain, which is calculated from $\varepsilon_{P}^{F} = D_{1} \left( {P* + T*} \right)^{{D_{2} }}$.

The JH-2 model implements the polynomial equation of state (EOS) defining the relationship between hydrostatic pressure *P* and volumetric strain $\mu$. For the intact material, the polynomial equation is given by the following^15,16,52,60^:

5 $$ P = K_{1} \mu + K_{2} \mu^{2} + K_{3} \mu^{3} $$

When the level of damage in the material increases, incremental pressure $\Delta P$ is added to the EOS. The value of this additional pressure changes from $\Delta P = 0$ at $D = 0$ to $\Delta P = \Delta P_{\max }$ at $D = 1$. The polynomial EOS with the added incremental pressure is described using the following formula^15,16,52,60^:

6 $$ P = K_{1} \mu + K_{2} \mu^{2} + K_{3} \mu^{3} + \Delta P $$

where $\mu = \rho /\rho_{0} - 1$ is the volumetric strain ($\rho$ is the current density, and $\rho_{0}$ is the reference density).

### Pressure constants

**Figure 2:**
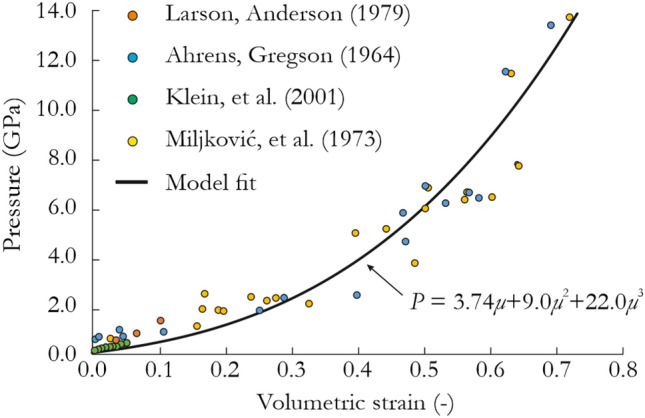
Visualization of fitting the JH-2 model EOS curve with literature shock test data for sandstone.

The methodology for determining the constants of the JH-2 model was presented in a previous study^61^. First, the pressure parameters, $K_{2}$, $K_{3}$ and $\mu$, were fitted to high-pressure impact experimental data for sandstone^62–65^ with the following constants: $K_{1} = 3.74$ GPa,$K_{2} = 9.0$ GPa, $K_{3} = 28.0$ GPa, providing a satisfactory fit to the given data as presented in Fig. 2.

Next, the Hugoniot Elastic Limit (HEL) was determined. Plate impact tests can be used to determine the HEL but were not performed for the studied sandstone. Instead, HEL values from the literature, i.e., 2.5 GPa^53^, 1.5 GPa^54^, 1.3 GPa^62^, 500 MPa^64^ and 4 GPa^66^, were averaged, and the result, HEL = 1960 MPa, was adopted for further calculations. Before determining the components of pressure *P*_*HEL*_ and deviatoric stress $\sigma_{HEL}$, the volumetric strain at the Hugoniot state was calculated as $\mu_{HEL} = 0.204$ using the following equation:

7 $$ HEL = K_{1} \mu_{HEL} + K_{2} \mu_{HEL}^{2} + K_{3} \mu_{HEL}^{3} + \frac{4}{3}G\frac{{\mu_{HEL} }}{{1 + \mu_{HEL} }} $$

Finally, $P_{HEL} = 1.37$ GPa and $\sigma_{HEL} = 0.91$ GPa were calculated based on Eq. (5) and the following relationship *HEL* = *P*_*HEL*_ + 2/3 $\sigma_{HEL}$.

### Strength constants

In the first step, the maximum hydrostatic tensile pressure *T* was calculated based on the spall strength in plate impact tests. Because plate impact tests were not conducted in this work, an average spall strength value of *T*_*spall*_ = 14.5 MPa for Nugget sandstone and Berea sandstone based on estimates by Grady and Hollenbach was adopted^67^. Notably, this value is similar to the dynamic uniaxial tensile strength obtained in the dynamic Brazilian test (please see Table 1). Subsequently, the normalized hydrostatic tensile pressure T* = 0.0058 was derived by fitting the constants *A, N* and T* from Eq. (1) to the HEL, data points from the SHPB tests, and the normalized components of spall pressure and deviatoric stress, which are described using the following equations, respectively:

8 $$ P_{spall}^{*} = \frac{{\frac{{T_{spall} }}{3}\left( {\frac{1 + \nu }{{1 - \nu }}} \right)}}{{P_{HEL} }} $$

9 $$ \sigma_{spall}^{*} = \frac{{T_{spall} \left( {\frac{1 - 2\nu }{{1 - \nu }}} \right)}}{{\sigma_{HEL} }} $$

Ultimately, the hydrostatic tensile pressure was calculated as $T = T^{*} /P_{HEL} = 8.0$ MPa.

Typically, the JH-2 model (Eq. 1) is fit to normalized experimental data of tensile and compression tests conducted under dynamic loading conditions^15,16,23,51,60^. However, the strain rate parameter *C*, which is fit using a simple one-constant logarithmic function to UC and SHPB tests and shock data, must be determined before this procedure. This approach omits lateral effects which will lead to an inaccurate representation of material dynamic strength. To address these effects, simulating the given SHPB tests utilizing the JH-2 model and adjusting the *C* constant to closely match the actual test results are recommended^56,68^. Thus, in this study, the strain rate effect parameter *C* was adjusted to obtain agreement between the SHPB test results and laboratory outcomes (see Section “SHPB dynamic uniaxial compression test”). Next, strength constants* A* = 0.71 and *N* = 0.55 were determined in a single-element UC test to achieve a compressive strength identical to that of the sandstone (see Section “Single-element tests”).

### Fracture strength and damage

Since it is challenging to empirically determine the constants used to reproduce the damage and fracture strength of sandstone, they were determined by trial and error. The parameters *B* and* M* were adjusted to achieve reliable results in the TXC structural test (Section “Triaxial compression test”), SHPB test (Section “SHPB dynamic uniaxial compression test”) and drop-weight impact test (Section “Drop-weight impact test”); ultimately, *B* = 0.3 and *M* = 0.4 were obtained*.* The maximum normalized fracture strength, σ*_max_ = 0.25, was considered to be the same as in^19^. Additionally, damage parameter values of *D*_*1*_ = 0.002 and *D*_*2*_ = 1.2 were chosen to enable an acceptable numerical reproduction of sandstone failure in all presented tests. Similar values were reported for dolomite^61^.

## Calibration and validation of the JH-2 model for sandstone

The JH-2 model with the parameters determined for sandstone was first examined using single-element tests, followed by structural simulations of quasi-static and dynamic tests characterized by different loading conditions and stress state complexities. All presented scenarios were simulated using an explicit LS-Dyna commercial hydrocode with multiparallel processing (MPP)^69–72^. Most models for the constitutive reproduction of brittle materials are mesh size dependent; therefore, conducting a mesh parametric study before the final numerical reproduction of the investigated test scenario is strongly advised. However, the influence of mesh size was not examined in the present study, as it was demonstrated previously that the value of maximum hydrostatic tensile pressure *T* in the JH-2 model should be adjusted depending on the element dimensions, especially if the tensile and shear deformation are dominant^56,61^. In most cases, an element size of 1.0 mm was adopted; when the mesh size was larger or smaller, *T* was adjusted according to previous studies. The JH-2 parameters that were ultimately determined for the sandstone are presented in Table 2.

**Table 2:** Material properties for the JH-2 constitutive model for sandstone.

Parameter	Value	Unit
Density, *ρ*	2350.0	kg/m3
Poison’s ratio, *v*	0.21	–
Bulk modulus, *K*_1_	3735.6	MPa
Shear modulus, *G*	2686.0	MPa
Elastic modulus, *E*	6500.0	MPa
Hugoniot elastic limit, *HEL*	1982.0	MPa
HEL pressure, *P*_*HEL*_	1374.0	MPa
Maximum tensile strength^a^, *T*	8.0	MPa
Intact strength coefficient, *A*	0.71	–
Fractured strength coefficient, *B*	0.30	–
Strain rate coefficient, *C*	0.022	–
Intact strength exponent, *N*	0.55	–
Fractured strength exponent, *M*	0.40	–
Bulk factor, *β*	1.0	–
Damage coefficient, *D*_1_	0.002	–
Damage coefficient, *D*_2_	1.20	–
Pressure coefficient 2, *K*_2_	9000.0	MPa
Pressure coefficient 3, *K*_3_	22,000.0	MPa
Maximum normalized fracture strength, *σ*^a^_*max*_	0.25	–

^a^Value should be adjusted based on mesh size.

In most cases, the numerical outcomes were compared with the experimental data obtained for at least five samples, shown in the figures as a shaded zone representing the area between the limiting curves obtained from experimental measurements. Furthermore, failure of the sandstone obtained from numerical simulations is shown in the figures using a fringe representing the damage index (*D* in Eq. 4). Once the damage reaches a value of 1.0, a fully damaged material is observed (a red curve in Fig. 1b).

### Single-element tests

**Figure 3:**
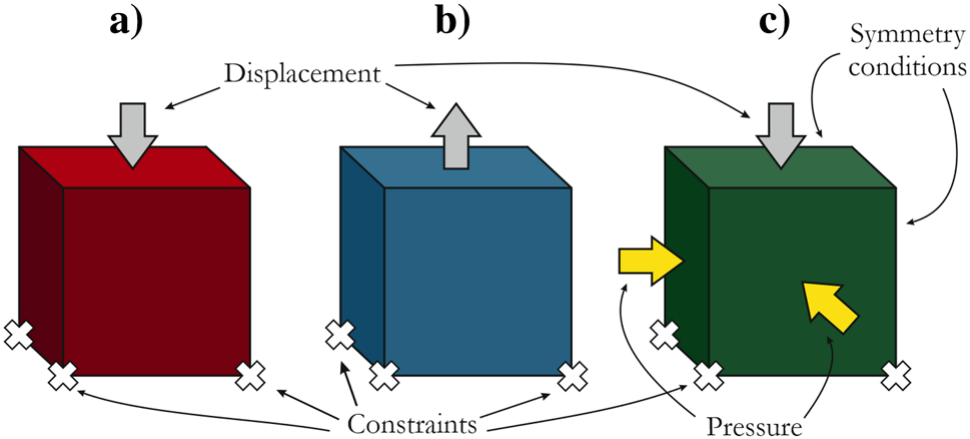
Boundary conditions for single-element tests for (**a**) quasi-static uniaxial compression test, (**b**) quasi-static uniaxial tension test and (**c**) quasi-static triaxial test.

A single cubic element with dimensions of 1.0 mm × 1.0 mm × 1.0 mm was adopted for the first-stage numerical simulations, which included the quasi-static uniaxial compression test (UC), quasi-static uniaxial tensile test (UT) and quasi-static triaxial compression test (TXC) (Fig. 3). The UC and TXC tests were also compared with the experimental data. The UT test was not conducted for the studied sandstone; therefore, only numerical results are discussed below.

**Figure 4:**
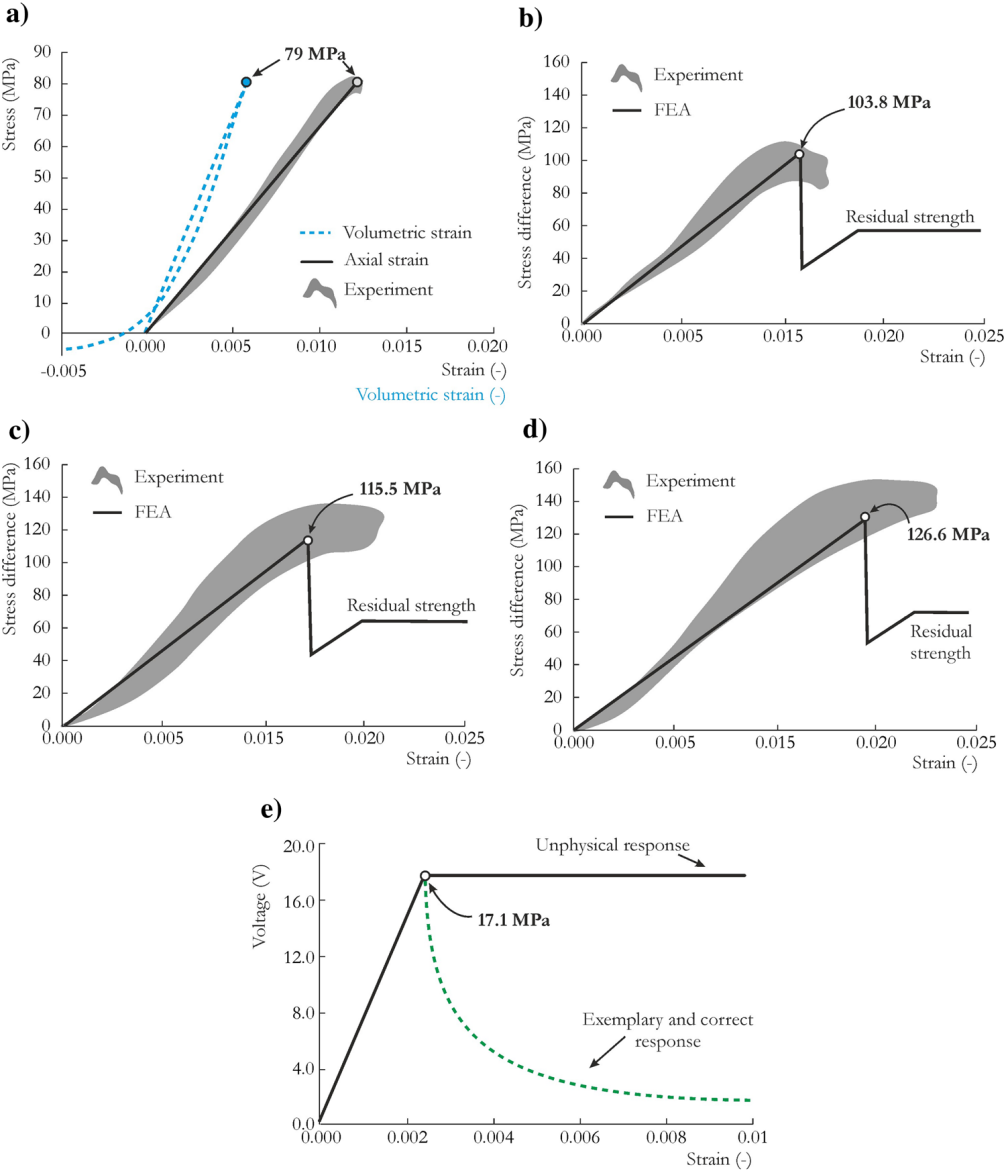
Comparison of the results from single-element numerical simulations and experimental tests for the determined parameters of the JH-2 constitutive model for sandstone: (**a**) UC test, (**b**) TXC test at 10 MPa confining pressure, (**c**) TXC test at 17 MPa confining pressure, (**d**) TXC test at 25 MPa confining pressure and (**e**) UT test.

The obtained stress vs. strain characteristics are presented in Fig. 4. The JH-2 model provided a satisfactory reproduction of the sandstone response under uniaxial compression loading. Nevertheless, since the JH-2 model is based on the *J*_*2*_ associative plastic flow rule, without the third invariant of deviatoric stress, the JH-2 constitutive model cannot correctly capture the shear dilatation from the material point of view. The presented volumetric strain response from the UC single-element test should not be interpreted as the actual shear dilatation of the rock material, as it is only a numerical effect at the material-point level. The JH-2 model cannot also stably reproduce stiffness degradation (softening) under uniaxial and triaxial loading conditions because the strength drops immediately after rock failure.

For the TXC tests, the comparisons of the stress vs. strain curves with the numerical reproductions based on the JH-2 model are presented in Fig. 4. As the confining pressure increased, the strength also increased; however, the JH-2 model did not reproduce strain hardening and strain softening, which were at nearly the same level as the increase in confining pressure. This limitation is due to the similarity of the softening paths for different confining pressures, which is mostly a result of normalization of *P* and $\sigma$ using $P_{HEL}$ and $\sigma_{HEL}$, respectively. A sudden drop from the maximum strength to the residual strength was observed and has no physical interpretation. The JH-2 parameters responsible for the fractured state of the material were taken as *B* = 0.3 and *M* = 0.4 so that a residual strength of 60% was obtained; similar results were reported by other scholars^73,74^.

For the UT test, the JH-2 constitutive model predicted elastic-perfectly plastic behavior, which is unphysical and not consistent with a proper response of brittle material under tension. This issue has been reported by several scholars^58,75^ and is one of the drawbacks of the JH-2 model. The remedy of this can be the application of user-defined models, which require implementing subroutine procedures^52,57,58^. Parallel studies demonstrated that by adding a very small shearing in the UT test the correct response of the material can be obtained. A pure tension is hard to occur in the loading scenarios presented in this paper, where a complex state of stress different than $\eta =1/3$ is more likely to be observed. Therefore, it was assumed that this limitation does not greatly influence the material behavior in the studied cases. Furthermore, the value of 17.2 MPa is significantly larger than the quasi-static tensile strength for sandstone (Table 1), as the JH-2 strength curve starts from T*, which according to the original papers by Johnson and Holmquist^15,16,52,60^, was taken from the flyer plate impact experiments (see Fig. 1a).

### Quasi-static structural tests

For the structural-level simulations, cylindrical specimens with a radius of 50.0 mm and a height of 100.0 mm were prepared with an element size of 1.0 mm. The specimens for the UC and TXC tests were inserted between two rigid walls: the lower was fixed, whereas the upper surface was able to move with the prescribed velocity according to previous studies^8,76,77^. Additionally, in the TXC test, pressure acting on the outer faces of the specimen was added, and the simulation was split into two stages: a dynamic relaxation phase to reproduce the hydrostatic compression and a compression loading phase, which was identical to the UC test.

#### Uniaxial compression test

When the UC test is conducted according to the ISRM standard, the radial shear stress can occur at the contact interface between the specimen and machine platens. This is due to significantly different mechanical properties of the platens and the specimen^78^. Moreover, the friction has a meaningful influence on the obtained cracking initiation and consequently failure pattern. Therefore, for sandstone and other rocks tested in UC obtaining repetitive and identical failure modes for numerous specimens is nearly impossible^6,8,78^; to solve this problem various testing configurations have been proposed^79,80^. Nevertheless, in the present paper, the basic experimental tests were conducted with ISRM configurations, thus, the above-mentioned problems occurred.

**Figure 5:**
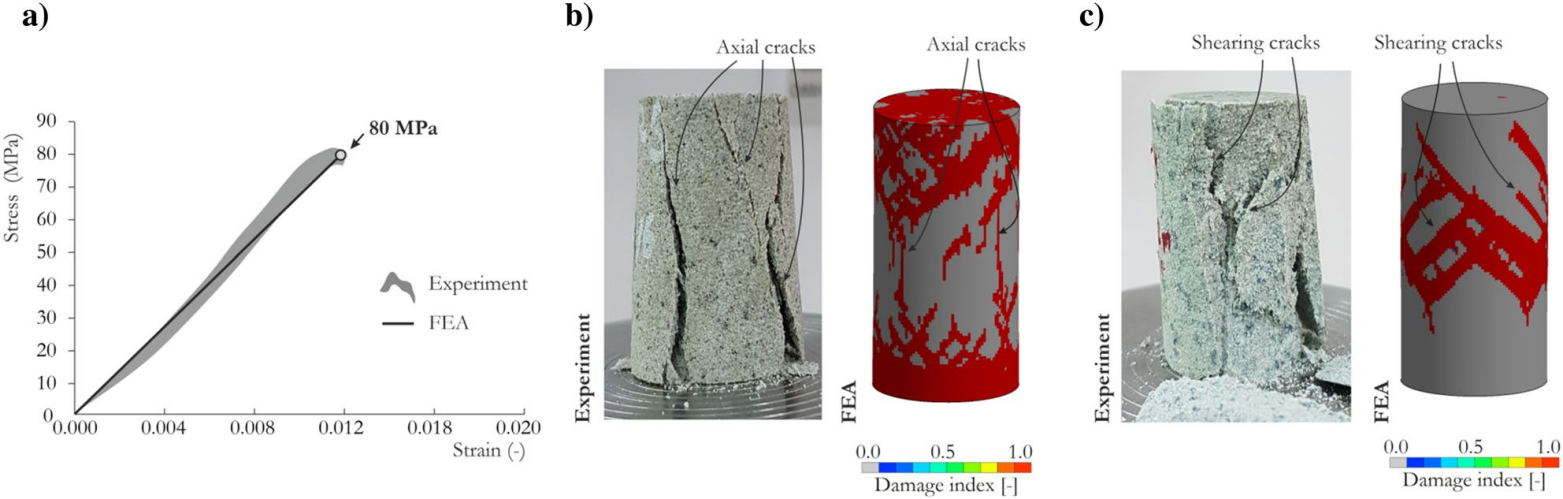
Structural UC test results using the JH-2 constitutive model for sandstone: (**a**) stress vs. strain curve; (**b**) AS failure pattern with corresponding FEM results without friction; and (**c**) MS failure pattern with corresponding FEM results with friction.

Figure 5a compares the experimental and numerical stress vs. strain curves for the UC test. A strength characteristic similar to that of the single-element test can be observed considering the maximum stress peak and the slope of the pre-peak curve. The corresponding failure patterns of the specimen are presented in Fig. 5b,c. During the experiments, two dominant failure modes were observed for the tested sandstone: axial splitting (AS) and multiple shearing (MS)^78^. Since the friction properties between the specimen contact surfaces and the surfaces of the specimens platens were unknown, the two numerical models were presented with (Fig. 5b) and without (Fig. 5c) friction between rigid walls representing the surfaces of the grip platens and the specimen. The implementation of friction had a pronounced effect on the observations and changed the failure mechanism from AS (without friction) to MS (with friction). Notably, applying friction had a negligible effect on the maximum strength (80.0 MPa with friction and 79.0 without friction).

In summary, these above-mentioned comparisons demonstrate that the JH-2 model with the parameters determined for sandstone provides a satisfactory reproduction of the specimen behavior in the UC test.

#### Triaxial compression test

**Figure 6:**
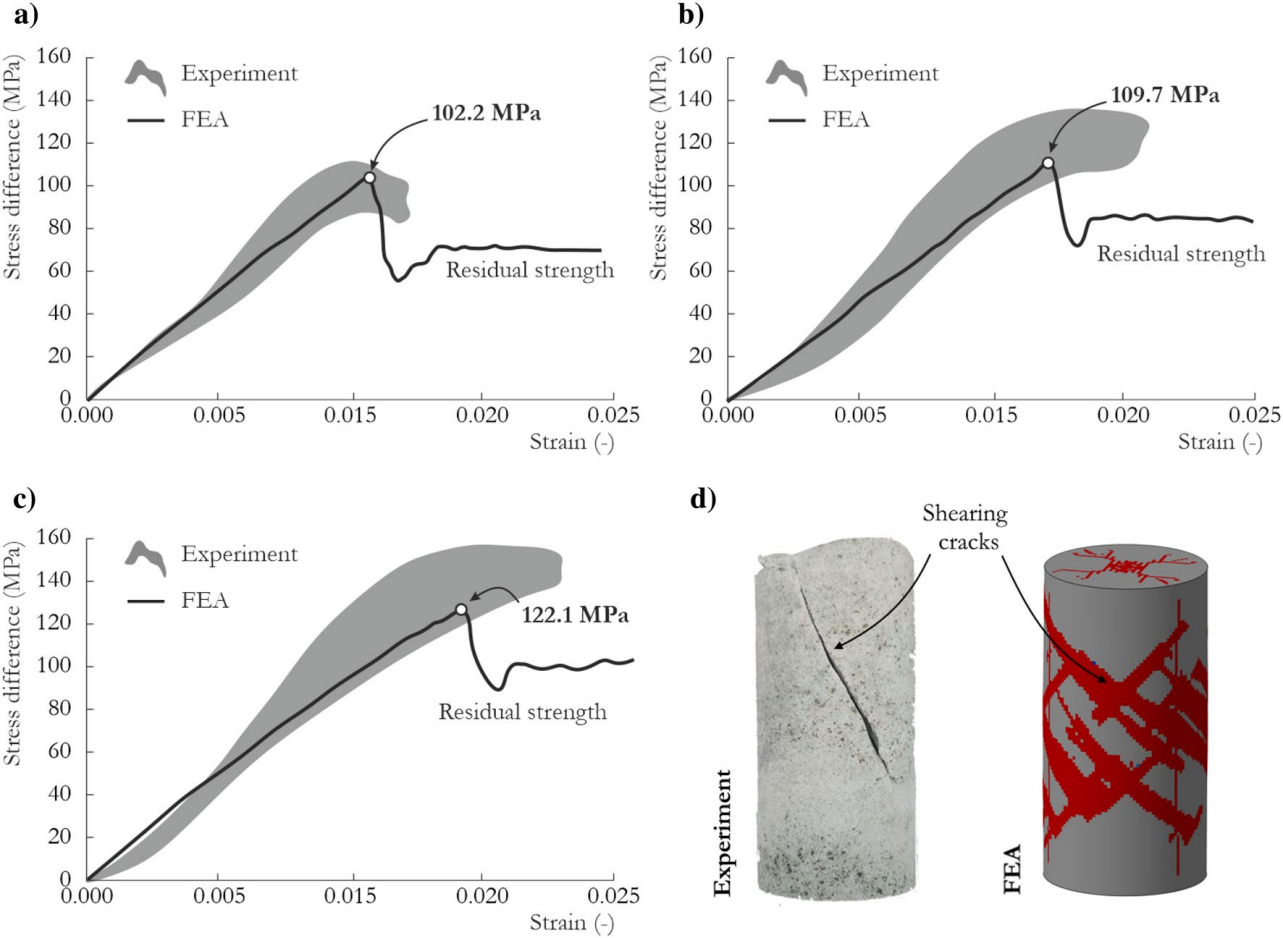
Structural TXC test results using the JH-2 constitutive model for sandstone: (**a**) 10 MPa confining pressure, (**b**) 17 MPa confining pressure, (**c**) 25 MPa confining pressure and (**d**) failure pattern with corresponding FEM results.

The experimental TXC curves and JH-2 model predictions for the sandstone under confining pressures of 10, 17.5 and 25 MPa are compared in Fig. 6a–c. The results from the experimental tests are shown as the shaded area, which represents the area between the limiting curves. The discrepancies between characteristics are a result of the heterogeneous structure of the sandstone and possible pre-cracks and inclusions in the specimens. The model satisfactorily reproduced the confined specimen’s behavior; however, the previously discussed drawbacks of the model, such as strain softening, were also evident at the structural level. The obtained residual strength was approximately 66% of the maximum strength, which is relatively high—60% was obtained in the single-element TXC test.

Notably, the maximum strength values obtained at each confining pressure were slightly smaller than the values from the single-element tests. These differences can be attributed to discretization, structural and boundary effects, friction, and damage calculations within the finite elements. The shear failure pattern predicted by the JH-2 model is presented in Fig. 6d, which also shows the real-world specimen after the test for comparison. In general, satisfactory agreement was obtained. Further validation against other laboratory tests showed that, despite the relatively high residual strength, the presented JH-2 model reproduced the experimental outcomes satisfactorily, as described in the next sections.

### Dynamic structural tests

#### SHPB dynamic uniaxial compression test

The SHPB apparatus comprised an air gun system; incident, transmitted and striker bars; velocity measurement system; and data acquisition system. The three bars were made of steel C45 and had diameters of 40.0 mm. One end of the incident bar was slightly truncated to have a diameter of 36 mm at the end to match the diameter of the striker. To minimize friction between the specimen and the contact surfaces of bars, a lubricant and polyester foil were used, which were proven to be very effective in previous papers^56,68^. It is worth noticing, that specimens were not under confinement during tests.

**Figure 7:**
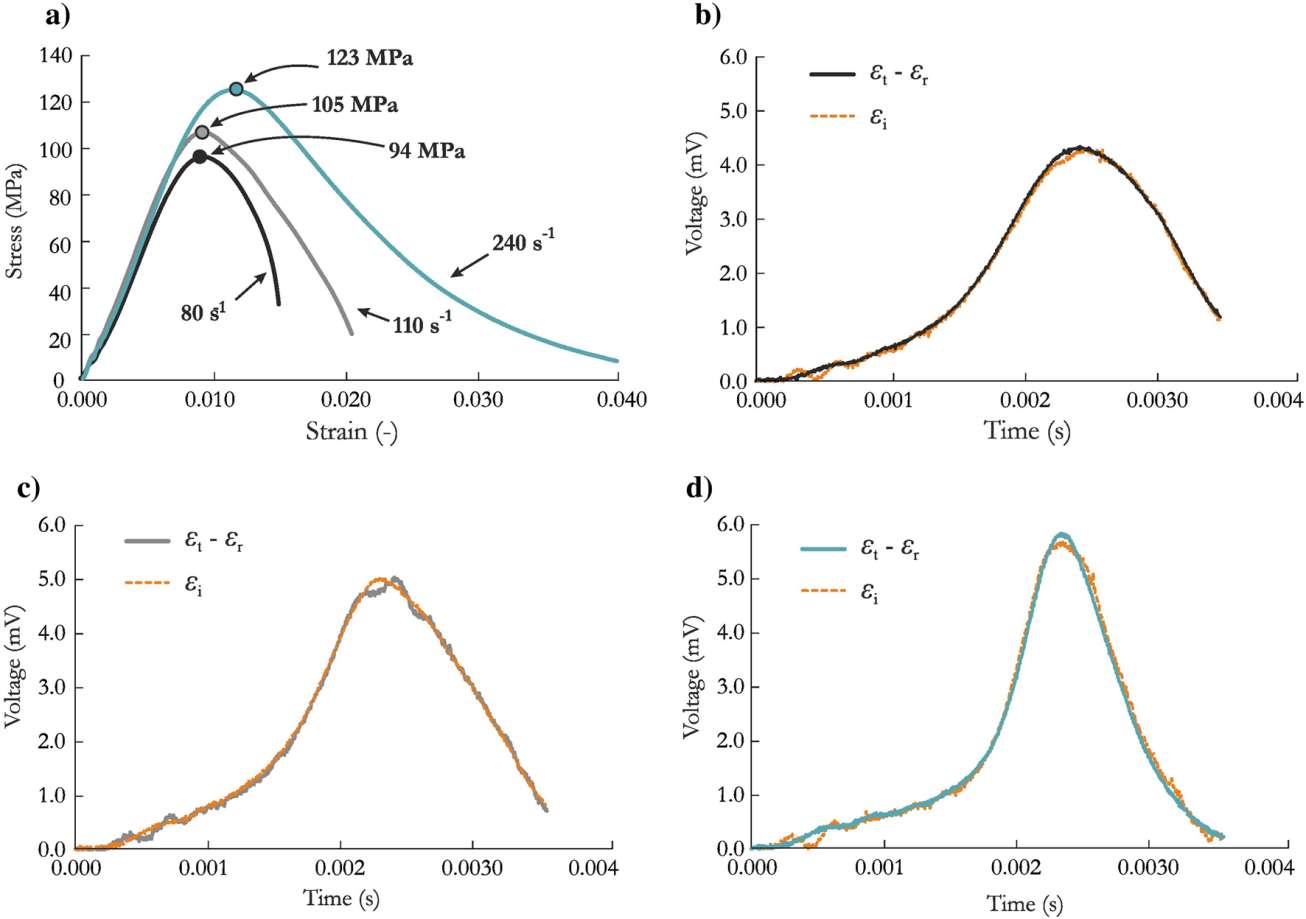
Dynamic compression response of SCC-90: (**a**) stress–strain curves; (**b**–**d**) equilibrium stress state for the specimen tested at strain rates of (**b**) 80 s^−1^, (**c**) 110 s^−1^ and (**d**) 240 s^−1^.

Performing the SHPB test with brittle materials demands the fulfillment of stress equilibrium and constant strain rate conditions during the dynamic compression process^81^. Only then, the results are valid and can be considered for further analysis or implementation in numerical simulations. Therefore, the profile of the incident wave during each test was modified by inserting copper pulse shapers between the striker and incident bar. Numerous tests were conducted for the investigated sandstone, and representative stress vs. strain curves are presented in Fig. 7a. Moreover, the verified stress equilibria for the three tests with strain rates of 80 s^−1^, 110 s^−1^ and 240 s^−1^ are shown in Fig. 7b–d. As the strain rate increased, a higher compressive strength was obtained. The two SHPB tests with the highest strain rates, 110 s^−1^ and 240 s^−1^, were selected for validation of the JH-2 constitutive model.

**Figure 8:**
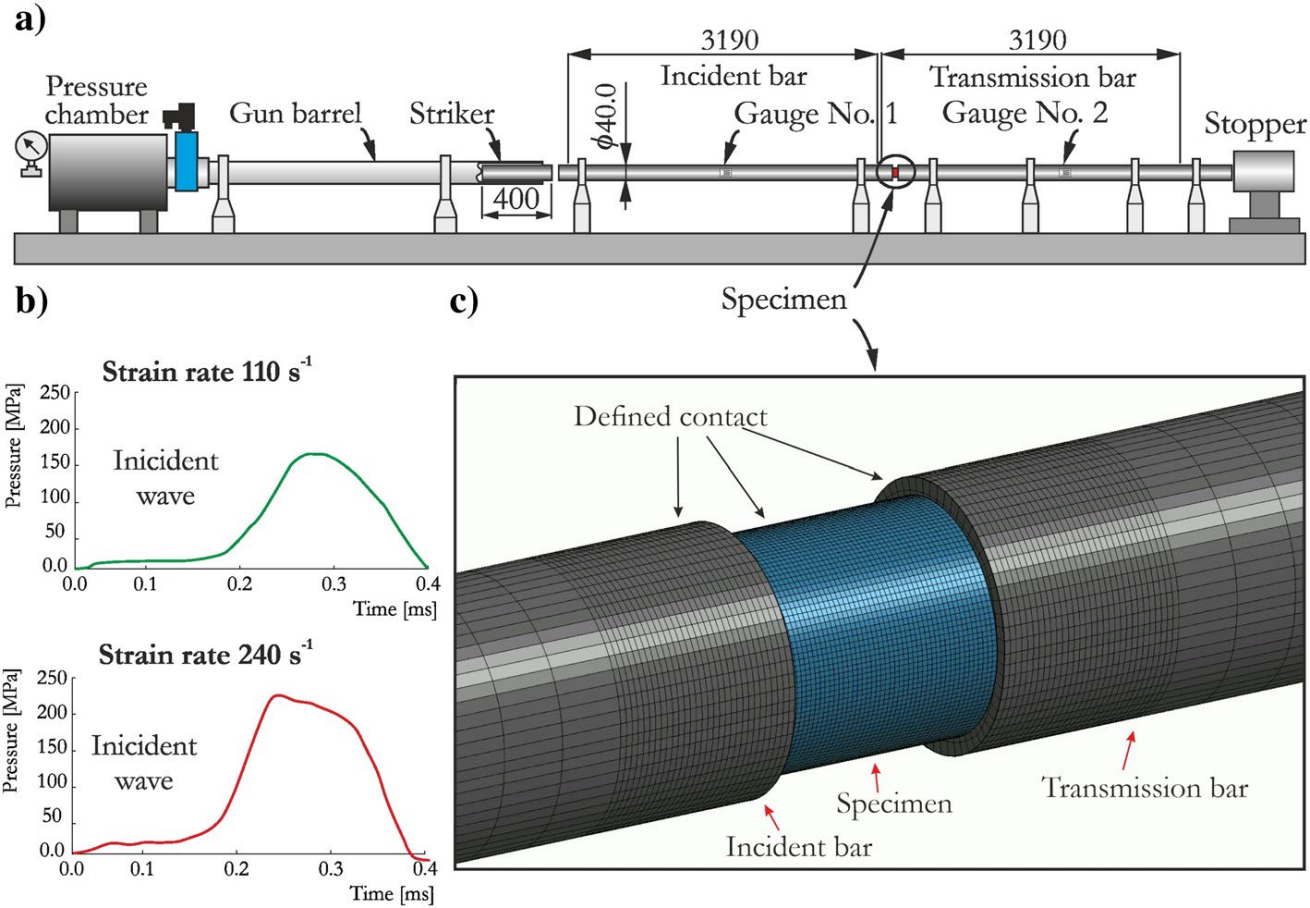
The SHPB setup used for dynamic uniaxial compression test of the sandstone: (**a**) scheme of the experimental SHPB apparatus, (**b**) incident pulses used for the simulations and (**c**) corresponding numerical model developed based on the real-world setup.

Numerical simulations of the SHPB test were carried out, and the FE model was developed based on the experimental setup presented in Fig. 8a. The striker was omitted from the model; thus, the incident wave was applied directly to the incident bar surface as a pressure load corresponding to the incident wave measured in the real-world tests (Fig. 8b). For the specimen and the two bars, brick elements were adopted; a finer mesh was used at both ends of the bars near the specimen. Ultimately, a mesh size of 1.0 mm was used for the specimen and 1.5 mm for the bar ends (Fig. 8c). The entire model consisted of 443,680 elements. An erosion technique can be used to visualize cracking as a loss of continuity in the specimen but was not introduced in this case. In previous studies^82,83^, it was demonstrated that when a lubricant is used in the SHPB experiment, a value of 0.1 or less can be considered for the friction coefficient in FEA, which eventually does not significantly influence the dynamic increase factor (DIF). In the present study, no friction was assumed in defining the penalty-based contact between the bars and the specimen since polyester foil and lubricant were used to minimize the end friction effect as much as possible in the laboratory investigations. The authors have effectively adopt this approach in previous studies^68,84^. The discussed numerical model is presented in Fig. 8b.

**Figure 9:**
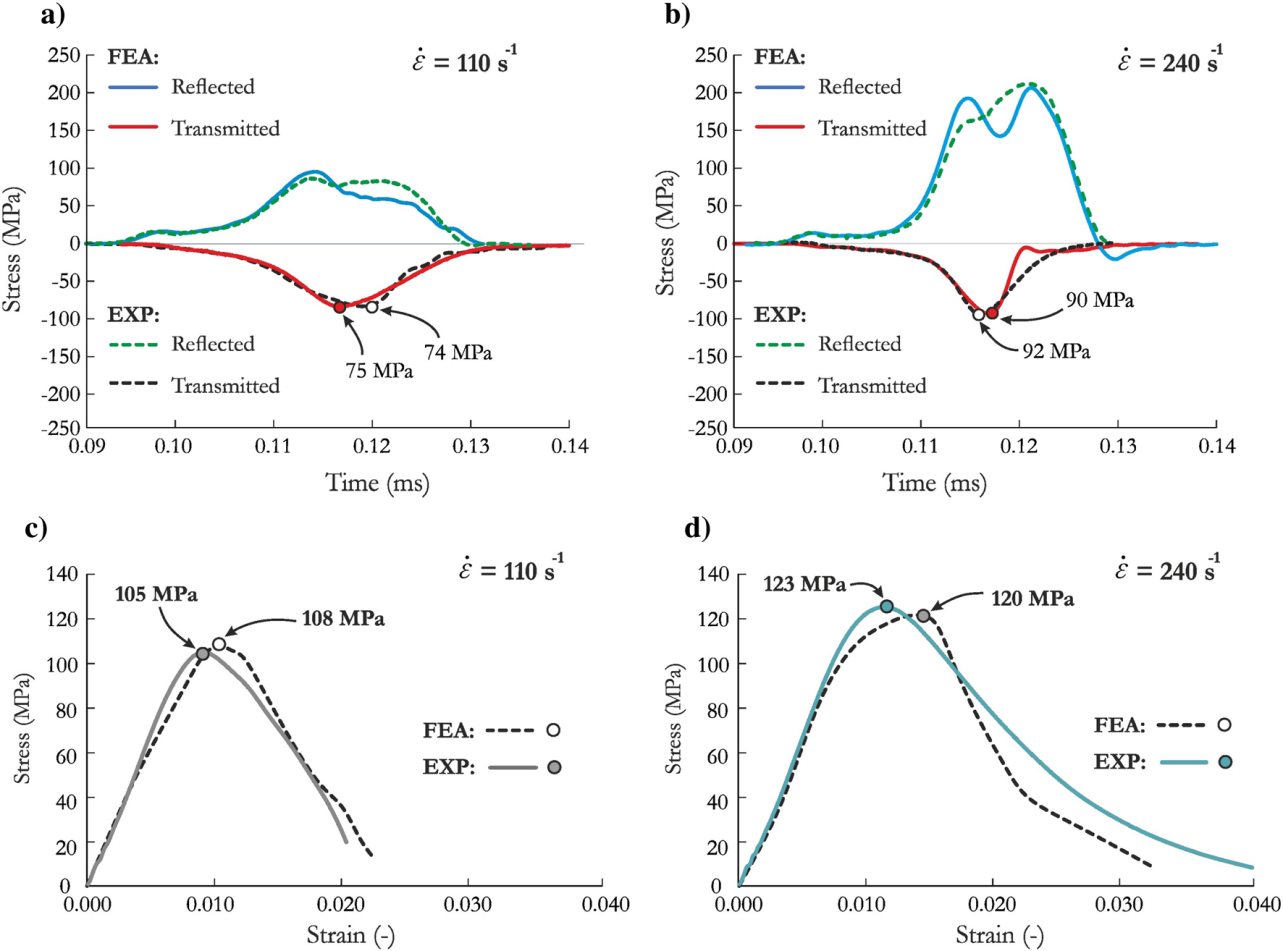
Comparison of waveform curves at a strain rate of (**a**) 110 s^−1^ and (**b**) 240 s^−1^ and stress–strain curves at a strain rate of (**c**) 110 s^−1^ and (**d**) 240 s^−1^ for FEA using the JH-2 model and experimental tests.

Figure 9a,b present the waveform data obtained in the experimental tests and finite element analysis (FEA) for the test with a strain rate of 110 s^−1^ and 240 s^−1^, respectively. In general, both curves were reproduced well by the JH-2 constitutive model. The detailed analysis and comparison presented here is based mainly on the transmitted curve, which reflects the stress vs. time relationship. The peak values for both tests were at most 2.2.% larger than the experimental values, which is expected since the strain rate coefficient in the JH-2 model was iteratively changed until the best agreement with the experimental data was achieved. The rise times were nearly identical to the laboratory outcomes. By contrast, the post-peak parts of the curves were not sufficiently similar to the experimental data, and a more brittle failure was observed in the case of higher strain rate. There are several potential reasons for these discrepancies. First, the FEM representation of the sandstone specimen treated the material as homogeneous, without microcracks, voids or inclusions. Second, the adopted approach did not represent cracks as a separation of elements, even when the erosion criterion was implemented. These observations are consistent with previous studies^56,68,85^. To address these problems, other approaches, such as peridynamics or smoothed particle hydrodynamics (SPH), may be more appropriate.

To further validate the JH-2 model, the stress vs. strain curves were also compared (Fig. 9c for a strain rate of 110 s^−1^ Fig. 9d for a strain rate of 240 s^−1^). In the FEA, the stress values were calculated from nodal forces and cross-sectional areas across the length of each specimen. The numerical stress–strain curves correspond well with the experimental outcomes. The JH-2 model provided an excellent match of the maximum strength value with the actual data, with an error not exceeding 3.0%. A very slight underprediction was obtained in the case with a strain rate of 110 s^−1^, whereas the maximum peak values were slightly overestimated for higher strain rate of 240 s^−1^. The post-peak part of the curve is not perfectly reproduced in both cases, especially in the case with a higher strain rate where more brittle failure was obtained in the numerical simulations compared with the experimental outcome. The differences in the post-peak curves are the result of the factors discussed earlier.

**Figure 10:**
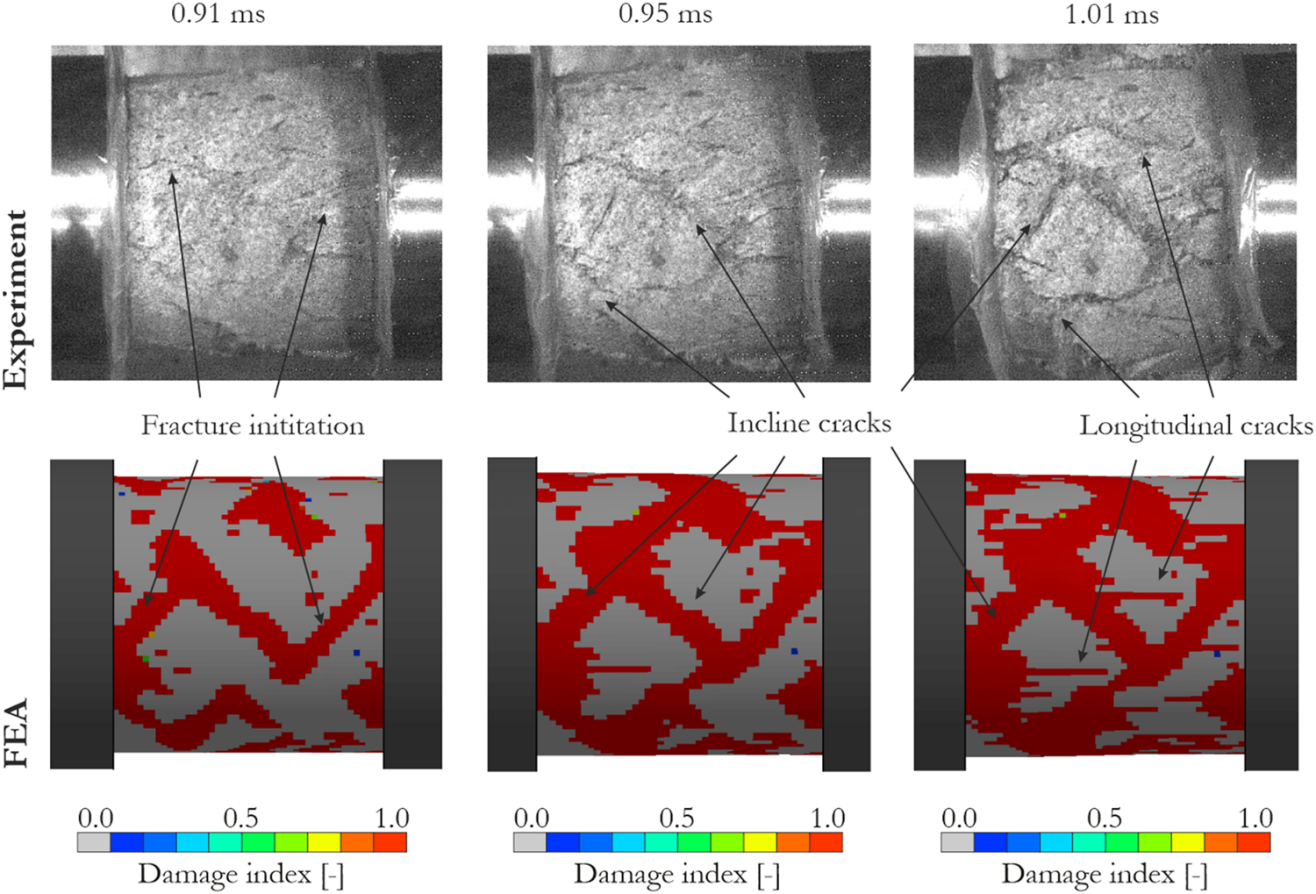
Comparison of specimen failure observed in the SHPB experiment and numerical simulations using the JH-2 model for sandstone.

A satisfactory reproduction of the waveform data and stress–strain curves presented earlier was also confirmed by quantitative comparison of the observed dynamic behavior of the specimen. An exemplary specimen failure in the three selected stages of the deformation process recorded with a high-speed camera is compared with the results of the numerical simulations in Fig. 10. The results are presented for the test with a strain rate of 110 s^−1^ because the deformation processes and material failure were similar in the three adopted cases. At the time of *t* = 0.91 ms, only few cracks propagated through the specimen which maintained its integrity, while at *t* = 0.95 ms several incline cracks were observed, and the specimen started to fracture. Eventually, at *t* = 1.01 ms, the load-carrying capacity of the sandstone was lost, and numerous longitudinal and incline cracks were formed. In both the experiment and FEA, similar results were observed proving that the JH-2 model reproduced well the cracking pattern. It also confirms that the damage parameters were correctly determined. A physical loss of sandstone continuity, especially visible at *t* = 1.01 ms, was not reproduced in numerical simulations since the erosion of elements was not implemented.

Despite the limitations of the model in capturing the softening phase of the transmitted and stress vs. strain curves, the model reflects the experimental SHPB tests in a satisfactory manner considering that the stiffness and strength enhancement had an excellent match with their counterparts. A qualitative comparison of the specimen behavior also demonstrated sufficient accuracy of the SHPB test predictions.

A similar consistence of experimental outcomes and numerical results in terms of quality and quantity have been reported in the previous findings made by other researchers^53,57,86^.

#### Drop-weight impact test

**Figure 11:**
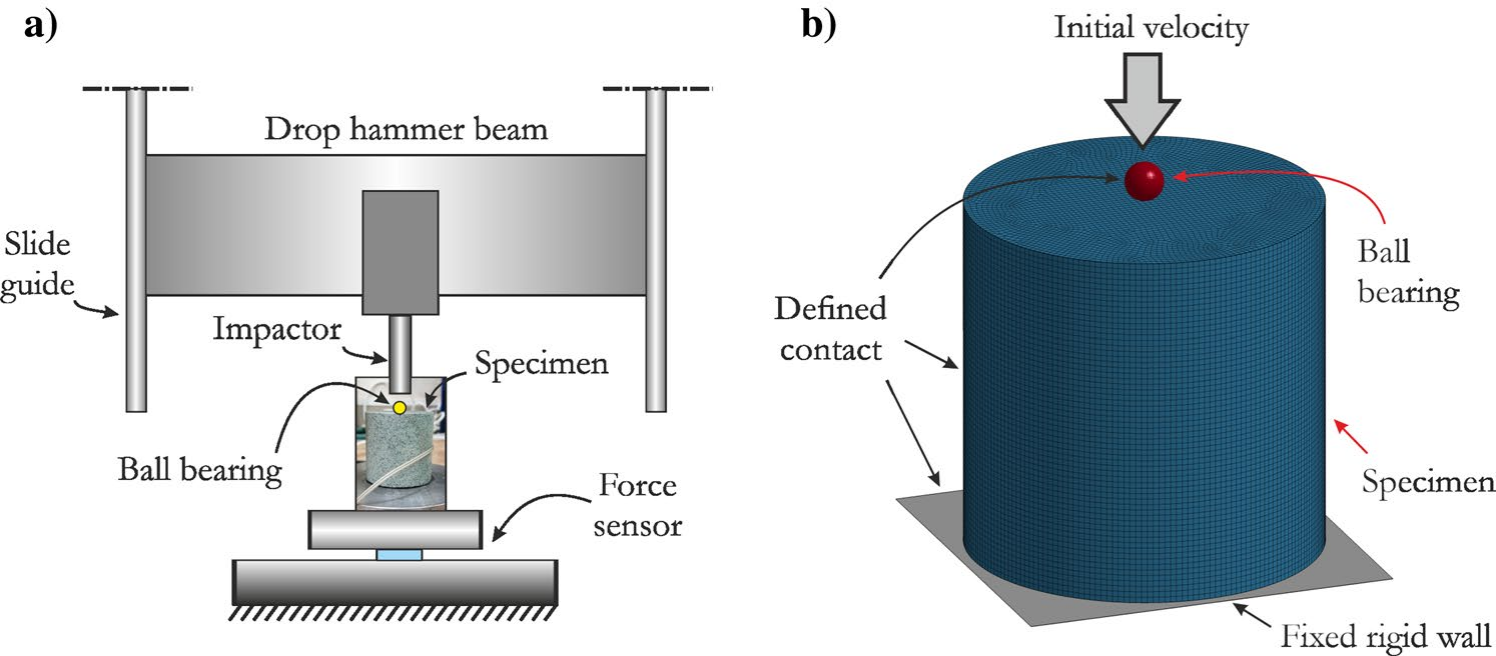
The dynamic drop-weight impact test of the sandstone: (**a**) scheme of the experimental setup and (**b**) corresponding numerical model developed based on the real-world setup.

The JH-2 model with the parameters determined for sandstone was further validated based on the drop-weight impact test using a ball bearing. This test was previously used to validate simulations of a dolomite rock^61^. A ball bearing with a diameter of 5.5 mm was placed on a cylindrical specimen with a diameter and height of 50.0 mm. The ball was impacted by a drop hammer beam with an average energy of 13.4 J. The experiment was simulated under the corresponding conditions, and for the sandstone specimen, hexagonal elements with a mesh size of 1.0 mm were employed. The ball was assumed to be rigid since no permanent deformation was observed during the laboratory tests. The impactor was omitted, and the mass of the ball was adjusted so that its impact energy was identical to that in the real-world experiment. For all interacting parts of the model, a penalty-based contact interface was used. Fig. 11 presents the scheme of the experimental setup and the numerical model.

**Figure 12:**
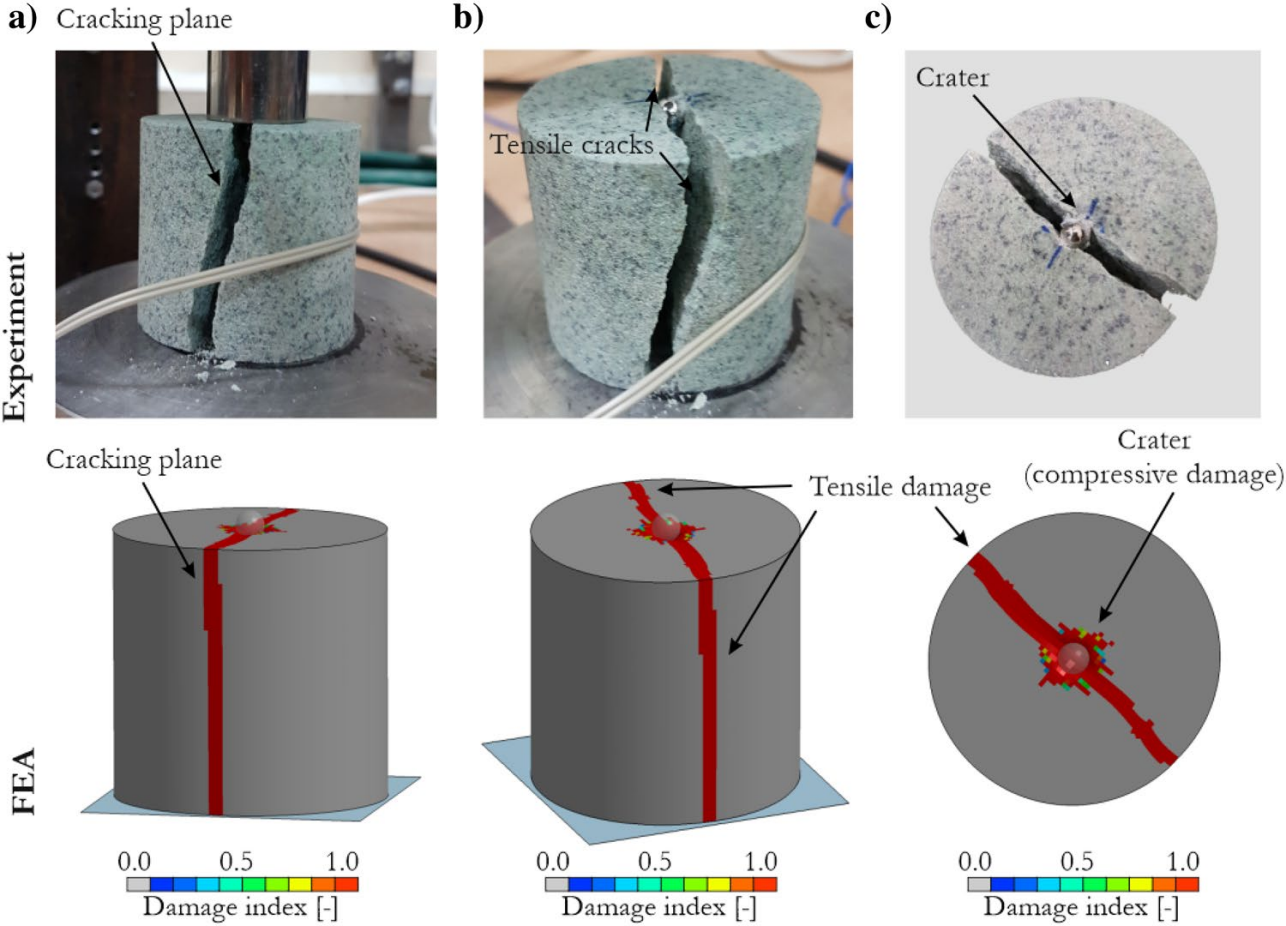
Comparison of specimen failure in the experiment and numerical simulations of the drop-weight impact test using the JH-2 model for sandstone: (**a**) front view, (**b**) isometric view, and (**c**) upper view.

The specimen failure obtained with the numerical model and JH-2 parameters for sandstone is presented in Fig. 12. The results are compared with photos of the real-world specimen, and three views are shown: (a) front view, (b) isometric view and (c) upper view. The compressive damage was localized in the area of direct contact with the ball bearing. A crater was formed under the steel ball, which initiated traverse cracks as it was further pressed into the specimen. Ultimately, the specimen split into two pieces. Excellent correspondence of the simulations with the real-world observations was observed.

**Figure 13:**
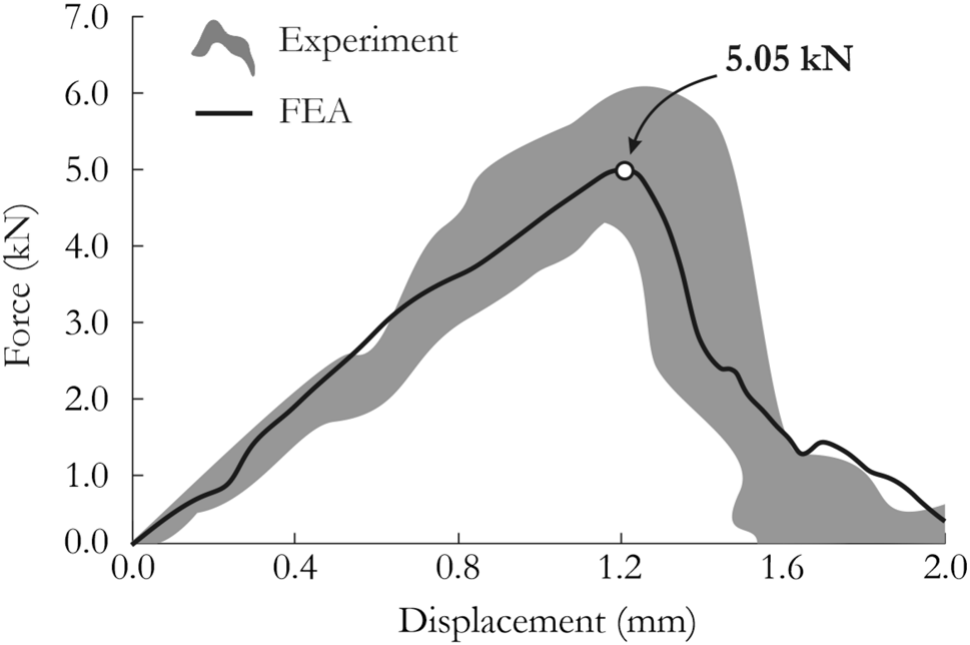
Comparison of force vs. displacement curves for the dynamic drop-weight impact test using a ball bearing obtained from experimental tests and FEA using the JH-2 model.

To quantitatively analyze the results, the force vs. displacement curves obtained from the numerical modeling were compared with laboratory measurements (Fig. 13). Since several experimental tests were conducted, the shaded area representing the scatter of the experimental curves is shown. The slope of the curve was similar to the actual force characteristic, and FEA slightly underestimated the force peak compared with experiments: 5.05 kN versus 5.12 kN. After peaking, the force dropped suddenly because of specimen splitting and fragmentation.

## Examples of JH-2 model implementation

The JH-2 model with the parameters determined for sandstone was further used to simulate two additional loading scenarios: a small-scale blasting test and a projectile impact test. The numerical outcomes were compared with real-world data to demonstrate the ability of the JH-2 model to predict material behavior for two different dynamic problems.

### Small-scale blasting test

Small-scale blast experiments were not performed with the investigated sandstone in the present study. Consequently, the numerical simulations of the small-scale blasting test conducted previously^56,87^ are described and compared with the analytical solution for calculating the number of radial cracks^88^. The adopted procedures follow a previous study^87^; therefore, only a brief description of the numerical modeling is provided here.

**Figure 14:**
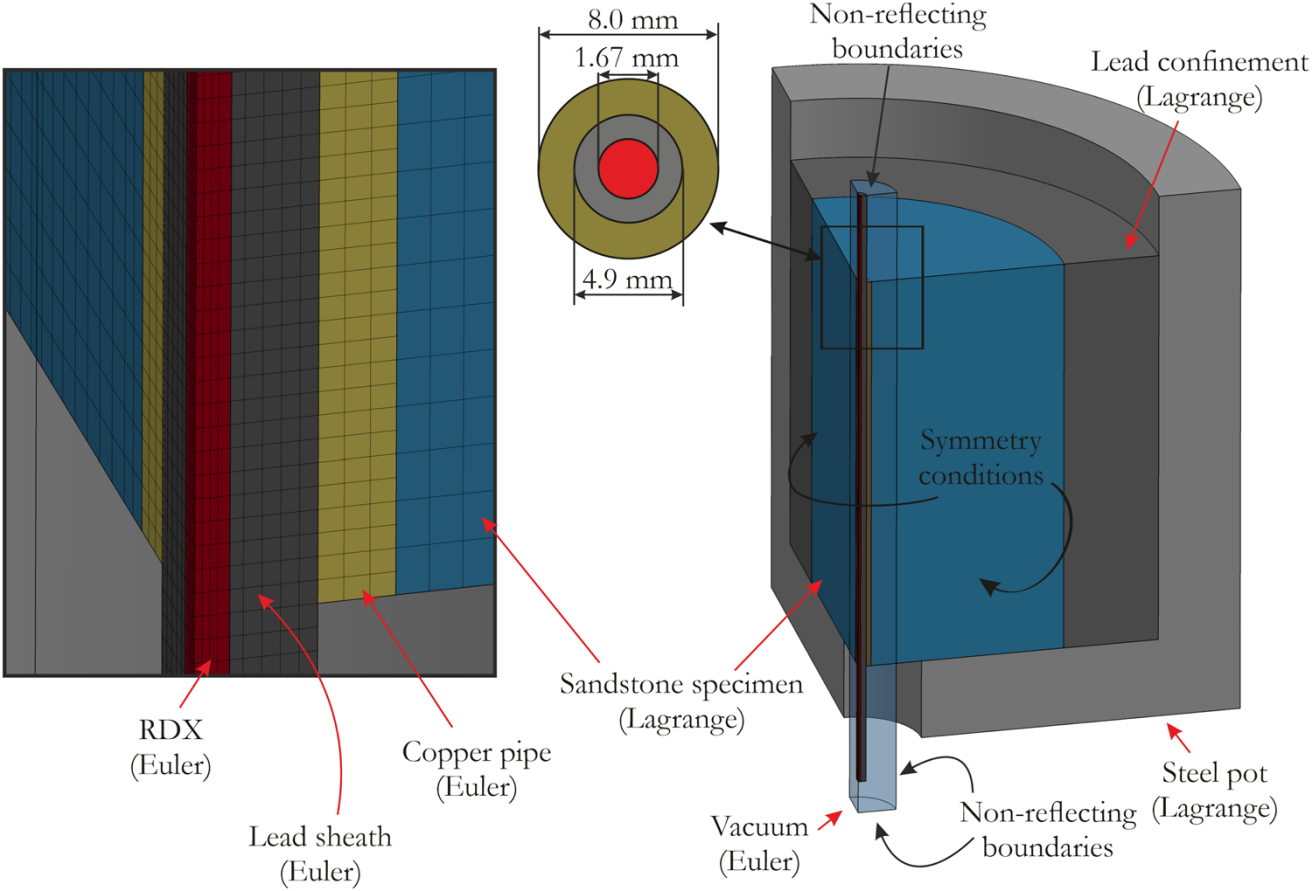
Schematic of the numerical model of the small-scale blast loading test.

A cylindrical specimen with a height and diameter of 130.0 mm was used in the numerical simulations. A borehole with a diameter of 8.0 mm was positioned in the center of the specimen, and a copper pipe with a wall thickness of 1.5 mm was installed in the borehole. Then, a detonation cord with the RDX HE covered with a lead sheath was placed inside the copper pipe (the RDX core had a radius of 0.7 mm). The specimen was confined using lead material to prevent extensive radial failure of the sandstone specimen. All parts were inserted in a steel pot with an inner diameter of 200.0 mm. The simulations were carried out using the 3D multimaterial arbitrary Lagrangian–Eulerian (MM-ALE) formulation method with a quarter model of the experimental setup. A finer mesh of 0.15–0.3 mm was used for the Eulerian components, whereas the sandstone specimen was modeled using a 1.0-mm mesh, resulting in a total of 997,445 elements. The parameters for the constitutive models used in the test can be found in an open access article^87^. The numerical modeling scheme is shown in Fig. 14.

**Figure 15:**
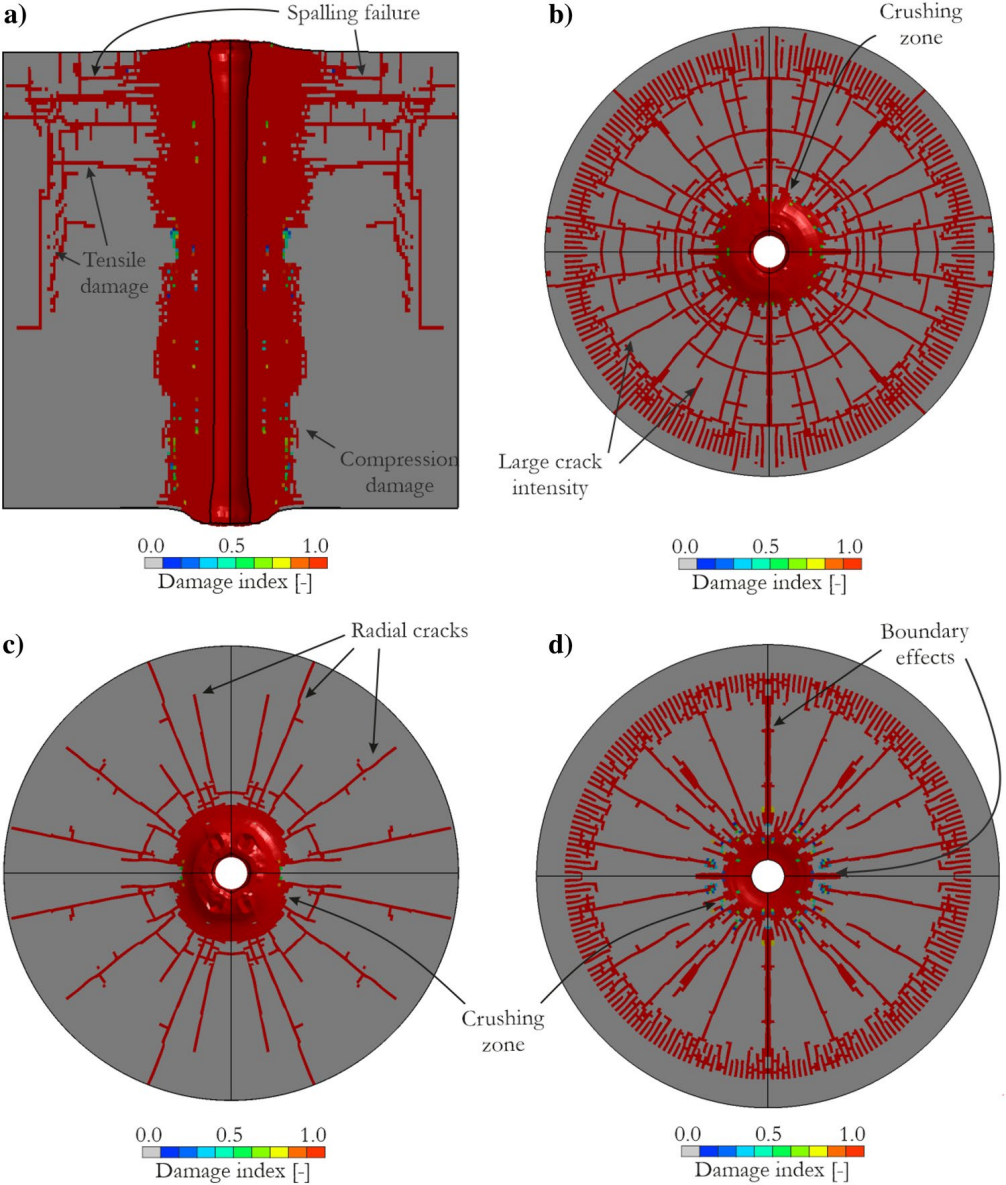
Blast-induced failure of the sandstone specimen: (**a**) side cross-section view; crack pattern in (**b**) top surface of the specimen, (**c**) bottom surface of the specimen and (**d**) middle cross-section of the specimen.

In Fig. 15, the sandstone failure resulting from the blasting process is presented. Fig. 15a presents a side view of the specimen showing the compressive damage close to the blast hole and the vertical cracks that were generated when the tensile strength of the sandstone material was exceeded. Furthermore, there was spalling damage at some distance from the top surface caused by the stress wave reflection from the surface. In Fig. 15b,c, the cracking patterns are shown for the top and bottom surfaces, respectively. The density of cracks was greater in the top surface than in the bottom surface. A surface at the middle cross-section of the numerical model is presented in Fig. 15d. This surface was used to further validate the JH-2 model by comparing the obtained radial cracks with the number of cracks predicted analytically based on fracture mechanics theory^88^. Notably, boundary effects resulting from symmetry conditions were observed, and the generated cracks were not considered in the comparison with the analytical formula.

The numerical model with the JH-2 constitutive model parameters determined for sandstone showed a good correlation with the analytical calculations. The number of cracks was similar for both methods, with a slight underprediction of the number of cracks in the numerical calculations: 24 cracks compared to 25 calculated using the formula shown in previous studies^87,88^. The comparison is presented in Table 3, which also includes the numbers of radial cracks in the upper and bottom surfaces. The outcomes were not compared with the real-world tests because such tests were not performed in the present work. The implemented small-scale blast setup was previously adopted for testing a dolomite rock^56,87^ and generated similar failure patterns and cracking characteristics, with certain differences resulting from the different properties of dolomite and sandstone. Similar failure patterns were observed in concrete^89,90^, sandstone^91,92^ and granite^19,93,94^.

**Table 3:** Comparison of the crack number obtained from the simulation and experiment.

Compared result		Experiment	Simulation	Theoretical
Number of radial cracks [−]	Top surface	–	28	25
	Middle surface	–	24	
	Bottom surface	–	20	

### Impact penetration test

**Figure 16:**
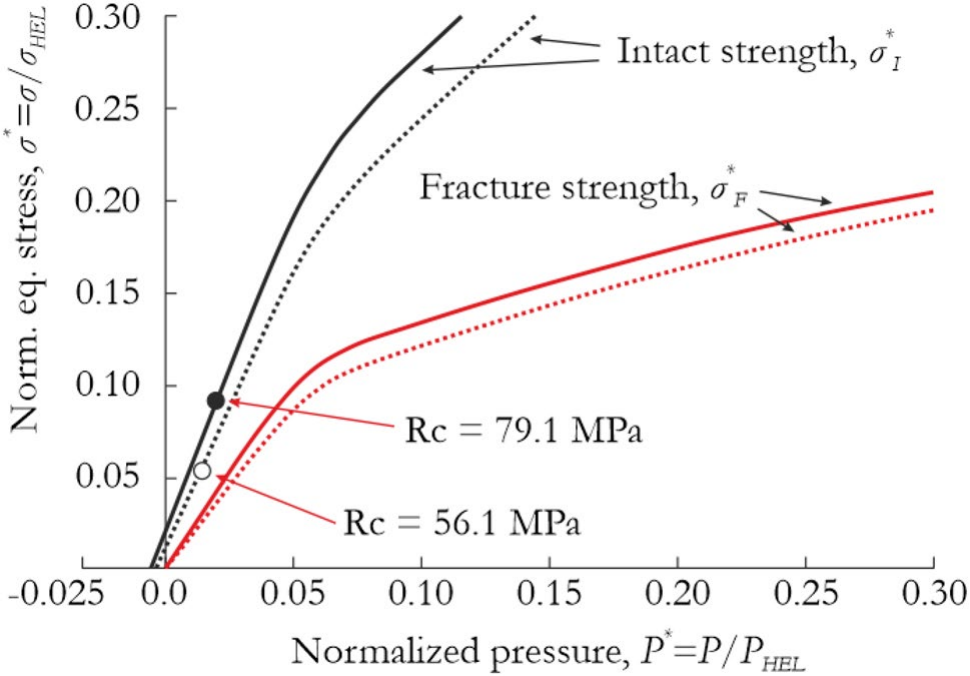
Comparison of the intact and fractured surfaces of the JH-2 constitutive model of the tested sandstone and the red sandstone used for projectile impact simulations.

To demonstrate the ability of the JH-2 model to reasonably reproduce impact dynamic loadings, a previously reported test of impact penetration of a projectile into a red sandstone target^43^ was simulated in the last stage. The properties of the red sandstone tested in the previous paper differ slightly from those of the sandstone investigated in the present paper. Therefore, based on the parameters reported by Zhang et al.^43^, the density, shear modulus and bulk modulus were changed to *ρ* = 2.575 kg/m^3^, *G* = 7.84 GPa and *K*_1_ = 10.51 GPa, respectively. Furthermore, the JH-2 parameters, i.e., *N* = 0.61 and *T* = 5.6 MPa, were adjusted to represent the uniaxial compressive strength of the red sandstone, i.e., *R*_*c*_ = 56.1 MPa, which was verified in a single-element test. Finally, the *M* = 0.44 parameter responsible for fracture strength was proportionally modified, whereas the other parameters were not changed. The JH-2 models for the investigated sandstone and red sandstone are compared in Fig. 16.

**Figure 17:**
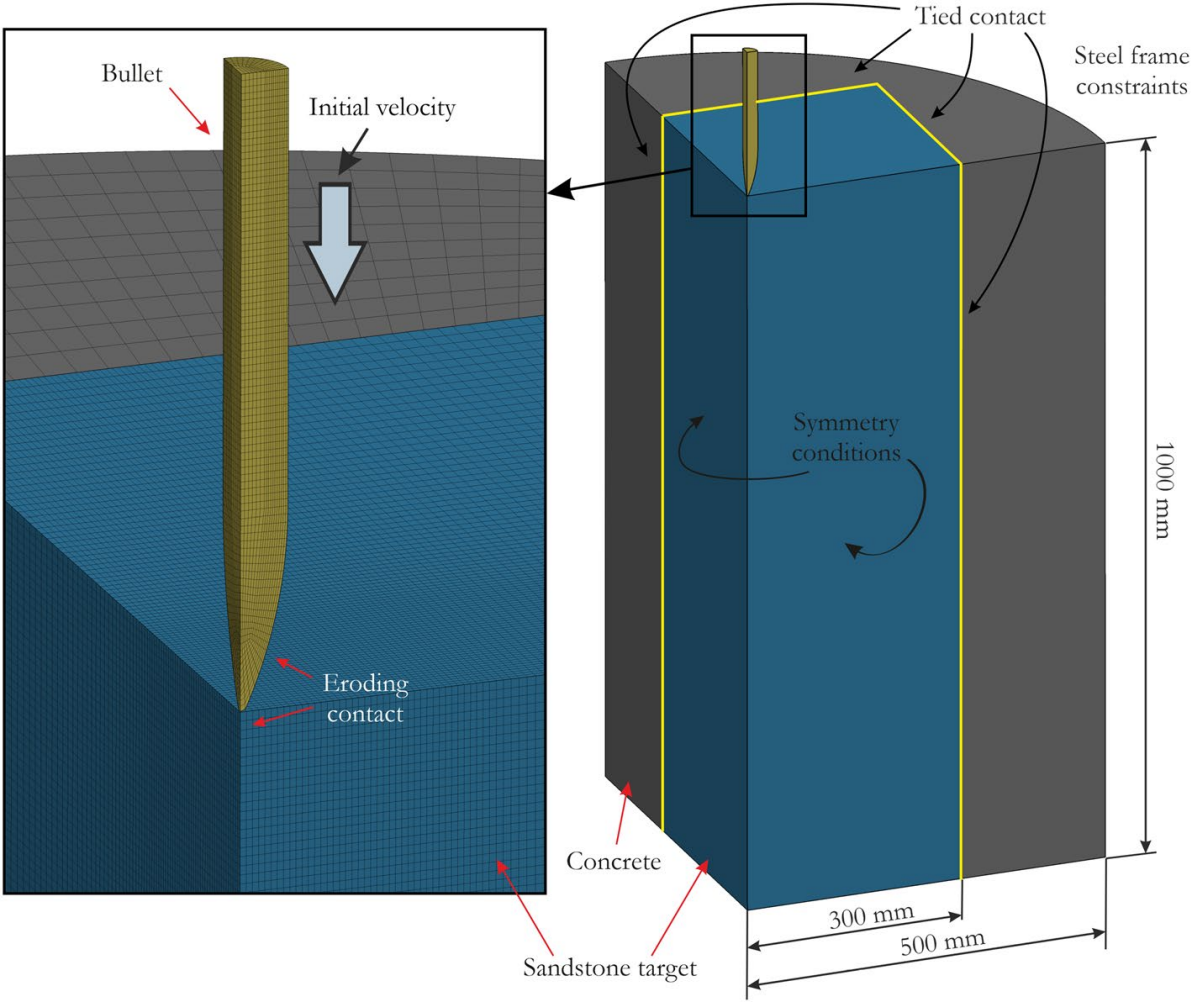
Numerical model of the sandstone target with the initial boundary conditions used to simulate the perforation test.

The numerical model was defined following Zhang et al.^43^. The sandstone target had a cuboid geometry with a square section of 600.0 mm × 600.0 mm and a height of 1000.0 mm. During real-world tests, the rock target was confined using C40 concrete, and a whole specimen was placed in a steel ring with a diameter of 1000.0 mm. Since the properties of the interface between sandstone and concrete are unknown, a kinematic constraint method was used to couple these two parts of the model. The concrete part was simulated using the automatically generated parameters of the KCC model for C40 concrete, and a non-deformable projectile was assumed. An average element size of 2.0 mm was adopted for the projectile and for the sandstone target directly interacting with the projectile (150 mm × 150 mm square). A quarter of the model was considered, with the appropriate boundary conditions on the relevant element faces. During the experimental tests, a steel frame was used to fix the targets during shooting. Therefore, the movement of all outer nodes of the concrete was constrained. To remove highly distorted elements and to simulate the failure of the sandstone target, element erosion, which is widely used in such problems^95–97^, was introduced. A value of 0.5 was used for the maximum principal strain (MXEPS). Although the adaptive transformation of Lagrangian elements into SPH particles can be used^98^, it was not implemented in the present paper because the intention was to develop an FE model as similar as possible to the model presented in^43^. Furthermore, a relatively large value of the erosion criterion was adopted to minimize the influence of mass loss and momentum on the results and simultaneously to achieve stable simulations. Friction was omitted because it is widely accepted to have a negligible effect^97,99^. Five projectile velocities were considered: 593 m/s, 700 m/s, 890 m/s, 900 m/s and 1000 m/s. The discussed model with the initial boundary conditions is presented in Fig. 17.

**Figure 18:**
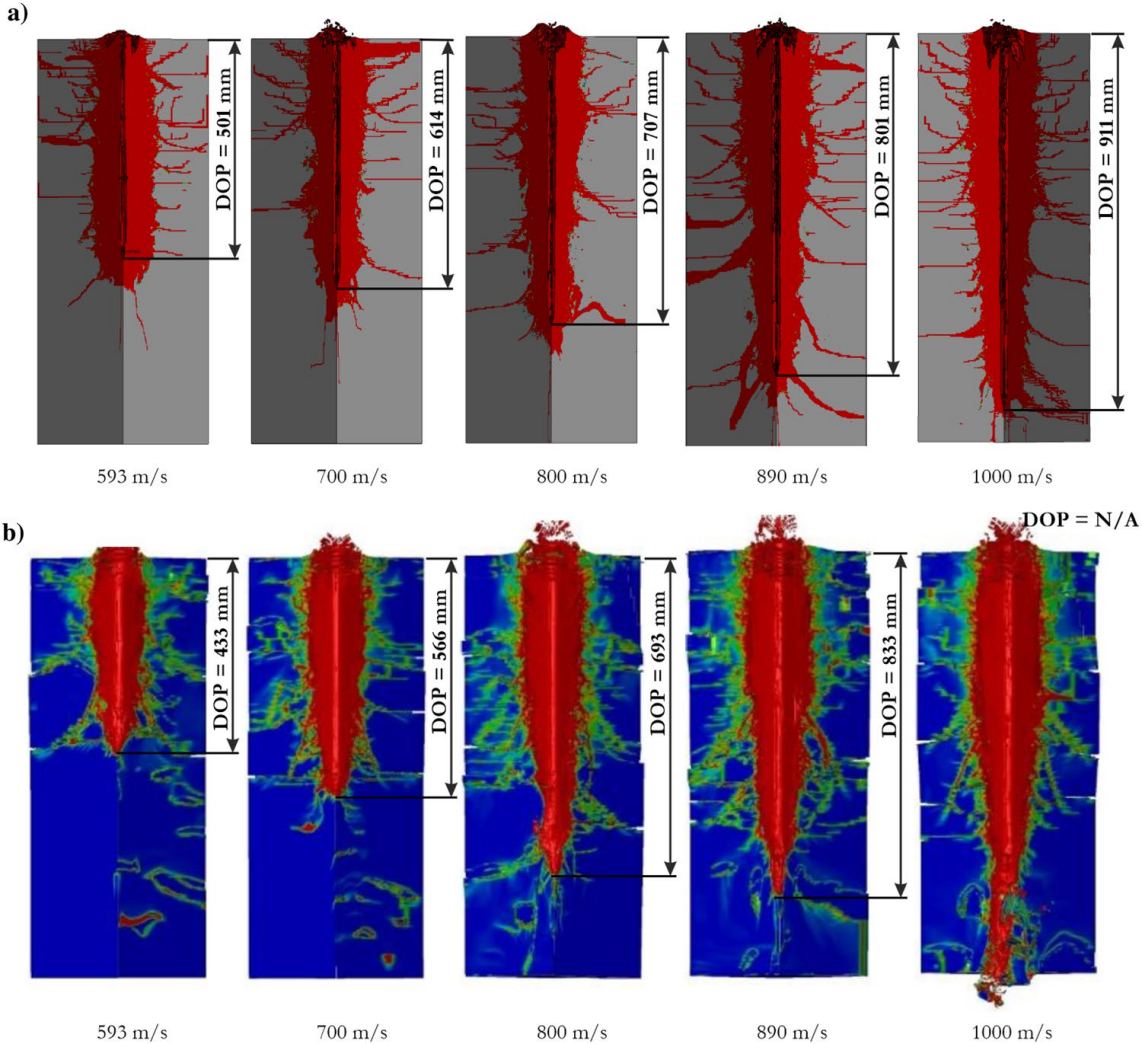
Comparison of specimen target failure due to projectile impact at investigated projectile velocities in the present study (**a**) and numerical simulations conducted by Zhang et al.^43^ (**b**).

The failure (in this case represented by the eroded elements and damage index) of the penetrated specimen at each of the projectile velocities is compared with the numerical simulations of Zhang et al.^43^ in Fig. 18. The impact of the projectile produced pronounced compressive/shear damage within the area of the impact, which was followed by the generation of transverse cracks from the center of the specimen to its outer surfaces. The damage patterns were generally consistent with the simulation results^43^ in terms of compaction area and obtained cracking. However, there were some discrepancies in the compression/shear zone width, which decreased toward the bottom of the sandstone target in the simulations^43^. Moreover, the change in this width was not as pronounced in the model as in the reference study. At the highest velocity (v = 1000 m/s), the spalling damage was significantly less pronounced than that obtained by Zhang et al.^43^.

**Figure 19:**
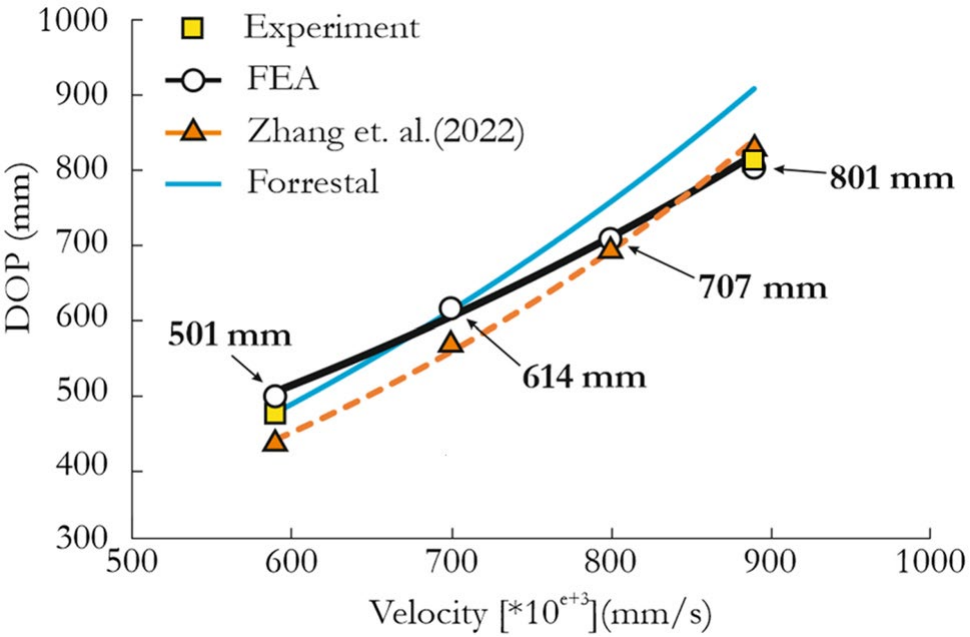
DOP vs. projectile velocity obtained from simulations using the JH-2 model and the results reported in^43^.

In Fig. 19 and Table 4, depth of penetration (DOP) vs. projectile velocity data are presented for the Forrestal formula^100^, JH-2 model and the experimental and numerical results of the reference study^43^. The JH-2 model overpredicted the DOP except at a projectile velocity of 890 m/s, for which it very slightly underestimated the DOP. On the other hand, the DOP values at *v* = 590 m/s and *v* = 700 m/s predicted by the present model are in satisfactory agreement with the results calculated using the Forrestal formula. The fit to the analytical calculations was worse at higher projectile velocities. Notably, the Forrestal formula did not match the trend of the data points from the experiment. This difference can be attributed to the specimen length of 1000 mm, which is very close to the DOP predicted for projectile velocities greater than 1000 mm/s. It appears that for a longer specimen the Forrestal formula would match the experimental and numerical results.

**Table 4:** Penetration depth data (in mm) obtained from simulations using the JH-2 model and the results reported in^43^.

Projectile velocity (mm\s)	Present study	Zhang et al.^43^		Forrestal formula	Error (%)		
	FEA^1^	FEA^2^	Experiment		FEA^1^/FEA^2^	FEA^1^/Exp	FEA^1^/Forrestal
590	501	433	470	463	15.7	6.6	8.2
700	614	566	–	614	8.5		0.0
800	707	693	–	782	2.0		− 9.6
890	801	833	810	901	− 3.8	− 1.1	− 11.1

There are several reasons for the discrepancies between the Forrestal formula, JH-2 model and the previous experimental and numerical results^43^. First, the interface conditions between the sandstone specimen and concrete confinement are unknown, and it is not clear whether Zhang et al.^43^ considered concrete in their model. Second, most of the implemented JH-2 model parameters were determined using the procedure presented in this paper. However, some were modified to match the compressive strength of the simulated red sandstone, and detailed calibration and validation of the parameters for this specific material are needed to obtain an ideal correspondence. The pressure, damage and strain-rate effect constants for the red sandstone may differ from those of the sandstone. Third, the erosion technique in which the removed Lagrangian elements are replaced with SPH particles should be used, as noted previously. Finally, the JH-2 model does not ideally represent the triaxial state of the material, as noted in Sections “Single-element tests” and “Quasi-static structural tests”, which is important for simulating projectile impacts. Nevertheless, the purpose of the analysis in this section was to demonstrate the JH-2 model’s ability to reproduce the results in a reasonable way without deeply analyzing its credibility and effectiveness following a detailed quantitative and qualitative approach.

## Conclusions

In this paper, the JH-2 parameters for a sandstone were determined by using an experimental and numerical methodology. On the basis of the results, the following conclusions can be drawn:

- The JH-2 model was originally proposed for simulating ceramic materials under dynamic loading, and the constants required for the specific material should be obtained from uniaxial high-strain-rate tests. As a result, the modelling of the quasi-static behavior of the material cannot reproduce the dilatation effect, has an unphysical tensile response, and cannot reproduce strain softening or the rapid drop in residual strength. These limitations were observed in single-element and structural tests.
- Despite the observed limitations and drawbacks of the JH-2 model, satisfactory reproduction of the quasi-static tests was obtained at the element and structural levels. The qualitative and quantitative comparison of FEA with the experiments confirmed an overall acceptable level of similarity.
- The simulations of dynamic problems proved that the JH-2 model with the determined parameters for sandstone was capable of reproducing the behavior of the sandstone in different stress states, with high convergence with the experimental results. However, to determine the strain rate effect parameter *C* in the JH-2 model, it is advisable to numerically reproduce the real-world SHPB tests to fine-tune the constant *C* and include lateral confinement, which can affect the strength enhancement of the material.
- In the last test, a few constants of the previously validated model were proportionally adjusted to match the compressive strength of the studied material, and credible simulations of the problem were obtained. Although certain discrepancies related to the specific material constants responsible for strain rate dependency, damage and material triaxiality reproduction were noted, the results were still at an acceptable level of agreement with the reference data.
- In summary, the presented JH-2 model with the determined and validated parameters can be effectively adopted for simulating other rock and brittle materials within several loading scenarios in the scope of quasi-static and dynamic regimes. It is important, however, to keep in mind the limitations of the model and its applicability, especially in quasi-static tests which are not dedicated to the JH-2.

## Data Availability

The datasets used and/or analyzed during the current study available from the corresponding author on reasonable request.
